# The pyroptosis-related gene signature predicts prognosis and indicates the immune microenvironment status of chronic lymphocytic leukemia

**DOI:** 10.3389/fimmu.2022.939978

**Published:** 2022-09-13

**Authors:** Yeqin Sha, Rui Jiang, Yi Miao, Shuchao Qin, Wei Wu, Yi Xia, Li Wang, Lei Fan, Hui Jin, Wei Xu, Jianyong Li, Huayuan Zhu

**Affiliations:** ^1^ Department of Hematology, The First Affiliated Hospital of Nanjing Medical University, Jiangsu Province Hospital, Nanjing, China; ^2^ Pukou Chronic Lymphocytic Leukemia (CLL) Center, Pukou Division of Jiangsu Province Hospital, Nanjing, China; ^3^ Jiangsu Key Lab of Cancer Biomarkers, Prevention and Treatment, Collaborative Innovation Center for Personalized Cancer Medicine, Nanjing Medical University, Nanjing, China; ^4^ National Clinical Research Center for Hematologic Diseases, The First Affiliated Hospital of Soochow University, Suzhou, China

**Keywords:** Chronic lymphocytic leukemia, pyroptosis, prognosis, immune microenvironment, gene

## Abstract

Chronic lymphocytic leukemia (CLL) is the most common leukemia in the Western world with great heterogeneity. Pyroptosis has recently been recognized as an inflammatory form of programmed cell death (PCD) and shares a close relationship with apoptosis. Although the role of apoptosis in CLL was comprehensively studied and successfully applied in clinical treatment, the relationship between pyroptosis genes and CLL remained largely unknown. In this study, eight differentially expressed pyroptosis-related genes (PRGs) were identified between CLL and normal B cells. In order to screen out the prognostic value of differentially expressed PRGs, univariate and multivariate Cox regression analyses were conducted and a risk model with three PRG signatures (GSDME, NLRP3, and PLCG1) was constructed. All CLL samples were stratified into high- and low-risk subgroups according to risk scores. The risk model showed high efficacy in predicting both overall survival (OS) and time to first treatment (TTFT). Gene Ontology (GO), Kyoto Encyclopedia of Genes and Genomes (KEGG), and Gene Set Enrichment Analysis (GSEA) showed the dysregulation of immune and inflammatory response in the high-risk group. Single-sample GSEA (ssGSEA) of immune cell infiltration and the activity of immune-related pathways also displayed decreased antitumor immunity in the high-risk group. In conclusion, PRGs are of prognostic value in CLL and may play important roles in tumor immunity, and the underlying relationship between PRGs and CLL needs to be explored further.

## Introduction

Chronic lymphocytic leukemia (CLL) is the most common leukemia in the Western world (1) with great heterogeneity. Owing to the increasing knowledge of the biological and genetic characteristics of CLL, a number of biomarkers were exploited and prognostic models with high efficacy were constructed. The international prognostic index for CLL (CLL-IPI) is one of the most well-recognized one, which incorporates TP53 status, immunoglobulin heavy chain variable region (IGHV) mutational status, serum _2_-microglobulin concentration, clinical stage, and age (2). The model discriminated CLL patients into four prognostic subgroups with 5-year overall survival (OS) ranging from about more than 90% in the low-risk subgroup to about 20% in the very-high-risk subgroup in both Western and Chinese cohorts (2–4). CLL-IPI was validated to be able to identify high-risk CLL patients who cannot benefit from conventional chemoimmunotherapies. However, whether novel, non-cytotoxic agents could overcome these inferior factors remains to be explored. Therefore, screening dysregulated pathways and aberrant gene expression in high-risk CLL is of great significance to understand the underlying mechanism of CLL progression.

Novel targeted agents including inhibitors of Bruton’s tyrosine kinase (BTK), apoptosis regulator B-cell leukemia/lymphoma 2 (BCL-2), and phosphatidylinositol-4,5-bis-phosphate 3-kinase catalytic subunit delta (PI3Kδ) have greatly changed the landscape in the treatment of CLL (5–7). Among them, venetoclax, which targets BCL-2 and the apoptotic pathway, is an effective and promising treatment option for CLL (8–10). Inhibition of anti-apoptotic BCL-2 proteins stimulates mitochondrial outer membrane permeabilization (MOMP) and leads to cytochrome c release and activation of a caspase cascade (11, 12). Notably, cysteinyl aspartate specific proteinase-3 (caspase-3) is a common key molecule in both apoptosis and pyroptosis pathways, which collaborates with gasdermin E (GSDME) as a switch between apoptosis and pyroptosis (13, 14). Although the mechanism of apoptosis underlying CLL is well-recognized and the BCL-2 inhibitors are broadly applied in clinical practice, dysregulation of pyroptosis underlying CLL has never been explored yet.

Pyroptosis is a novel form of programmed cell death (PCD) with characteristics of cellular swelling, lysis, and the release of many inflammatory factors (15) while apoptosis a non-inflammatory PCD. The gasdermin family is the main candidate of pyroptosis and activates pyroptosis through two main approaches (1): gasdermin D (GSDMD)-dependent activation regulated by caspase-1/4/5/11, and (2) gasdermin E (GSDME)-dependent activation regulated by caspase-3 (16–19). Chemotherapeutic agents such as paclitaxel, cisplatin, and doxorubicin could induce pyroptosis in order to inhibit tumor progression and certain drugs might evoke the switch from caspase 3-dependent apoptosis to pyroptosis (20–23). Therefore, the role of pyroptosis underlying CLL, especially in the treatment of venetoclax or other chemotherapy, remains to be explored. Moreover, release of inflammatory cytokines (IL-1β, IL-18, and IL-16) and alarmins (ATP, HMGB1) might be immunogenic, recruiting and activating immune cells in the tumor (24, 25). Whether the effect of inflammation is tumor-promoting or inhibitory depends on the biological features of the particular tumor and remains to be explored.

In the present study, we obtained pyroptosis-related genes (PRGs) differentially expressed between CLL and normal B cells. PRGs related to prognosis were systematically screened out through univariate and multivariable Cox regression analysis, and the prognostic model was established. The model was further validated and patients were divided into high- and low-risk subgroups according to PRG model scores. Dysregulated pathways and immune cell infiltration between two subgroups were compared. Moreover, the expression of PRGs with prognostic significance was explored between IGHV mutated and unmutated CLL patients from Gene Expression Omnibus (GEO) datasets and our real-world treatment-naïve (TN) CLL cohort, and the efficacy of our PRG model was also validated in CLL patients of our center.

## Materials and methods

### Data acquisition and processing

**Figure 1:**
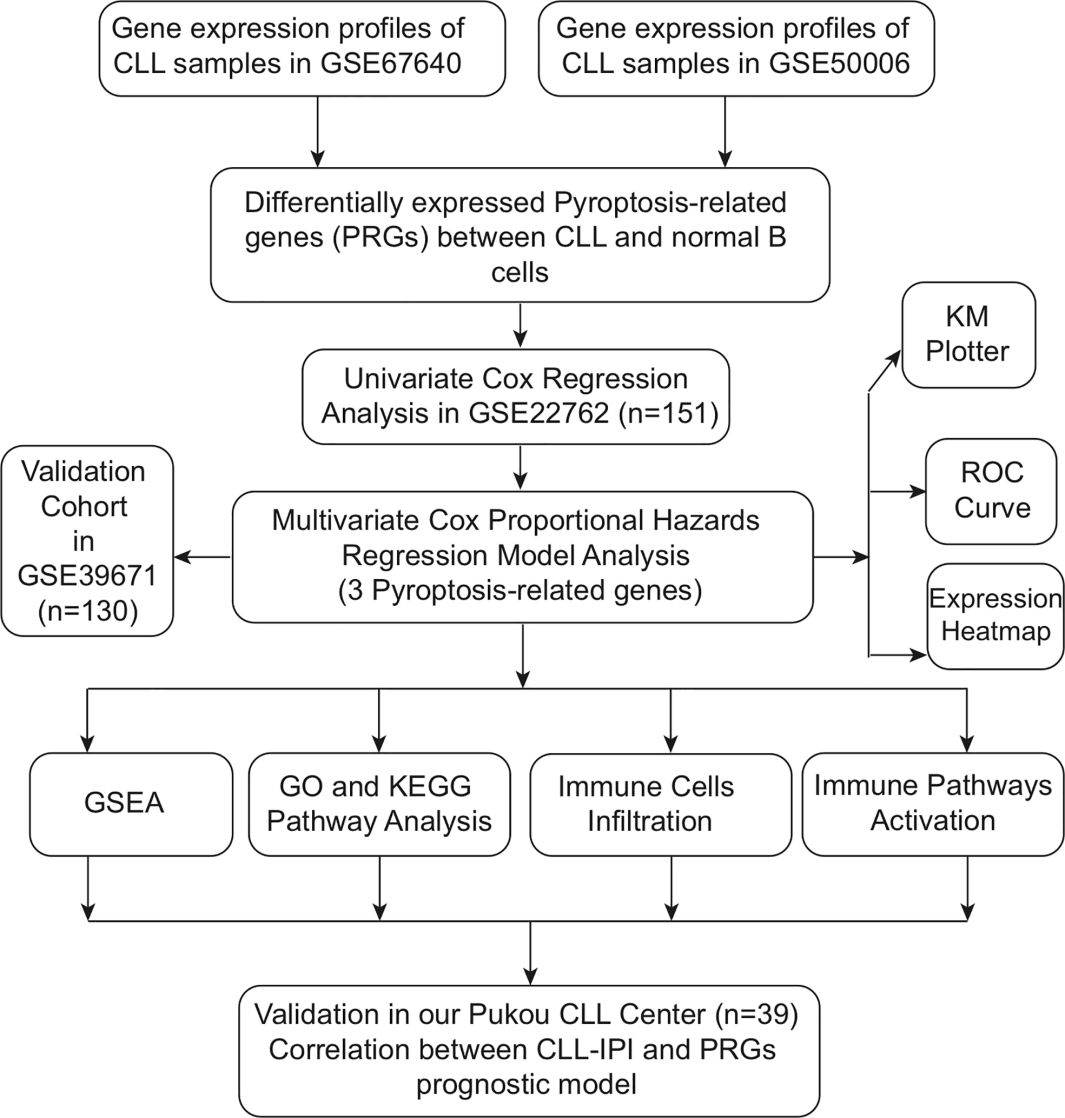
Flowchart of this study.

The public expression matrix and corresponding clinical data were obtained from the GEO database (https://www.ncbi.nlm.nih.gov/geo/). The details of GEO datasets used in our study are summarized in **Table 1** (26–30). Samples of GSE67640 datasets obtained from 15 CLL patients and nine healthy donors were applied to screen the differentially expressed PRGs (26). GSE50006 datasets including 188 CLL samples and CD19+ B cells from 32 healthy donors were used to validate the screened differentially expressed PRGs. GSE22762 included microarray datasets of 151 CLL peripheral blood mononuclear cell (PBMC) samples (44 Affymetrix HG-U133 A&B and 107 Affymetrix HG-U133 Plus 2.0 chips) to identify PRGs associated with OS; among them, 101 samples were used to validate PRGs related with time to first treatment (TTFT) (27). Normalization by normalizeBetweenArrays in the *limma* package is conducted for a better combination of data from two platforms, and combat algorithm in the sva package was used to correct intra- and inter-batch effects (31). At data cutoff, 41 CLL patients from the 151-CLL-sample cohort were dead and 56 of 101 patients received treatment. Treatment options of CLL patients included purine analogs, alkylating agents, bendamustine, rituximab, and alemtuzumab, while no patients received novel agents as treatment options. GSE39671 including microarray datasets of PBMC obtained from 130 TN CLL patients was used to validate PRGs associated with TTFT (28). GSE38611 and GSE51528 used purified CLL cells so that bias caused by PBMCs could be checked. Moreover, RNA samples of purified CLL cells from 39 TN patients in our center were obtained in order to validate the clinical and prognostic significance of PRGs. The detailed flowchart of this study is displayed in **Figure 1**.

**Table 1:** The summary of GEO datasets used in the present study.

GEO accession	Platform	Samples	Patient subgroup	Application
GSE67640 (26)	GPL10558	24	15 CD19+ cells from CLL patients and nine normal B cells from healthy donors	Selection of differentially expressed PRGs between CLL and normal B cells
GSE50006	GPL570	220	188 CLL samples and 32 normal B cells from healthy donors	Validation of differentially expressed PRGs between CLL and normal B cells
GSE22762 (27)	GPL96/GPL97/GPL570	151	151 CLL samples	Construction of a PRG prognostic model and survival analysis of PRG expression (OS and TTFT).
GSE39671 (28)	GPL570	130	130 CLL samples	Validation of a PRG prognostic model and survival analysis of PRG expression (TTFT).
GSE51528 (29)	GPL6244	216	131 M-CLL and 85 U-CLL	Validation of differential expression of selected PRGs between M-CLL and U-CLL.
GSE38611 (30)	GPL6244	136	76 M-CLL and 60 U-CLL	Validation of differential expression of selected PRGs between M-CLL and U-CLL.

GEO, Gene Expression Omnibus; PRGs, pyroptosis-related genes; OS, overall survival; TTFT, time to first treatment; M-CLL, immunoglobulin heavy chain variable region (IGHV) mutated CLL; U-CLL, IGHV unmutated CLL.

### Identification of differentially expressed PRGs

Thirty-three PRGs were extracted from prior reviews and present in **Table S1** (15, 32, 33). The “*limma*” package was used to identify differentially expressed genes (DEGs) between CLL cells and normal B cells with adjusted *p* value < 0.05 and absolute value of fold change (FC) ≥ 1.5. The cohorts from GSE67640 and GSE50006 were used and the intersection of upregulated and downregulated PRGs was obtained to conduct the following analysis. The differentially expressed PRGs were loaded into Metascape (https://metascape.org/gp/index.html#/main/step1) to conduct functional analysis and into Search Tool for the Retrieval of Interacting Genes (STRING, https://string-db.org/) to construct a PPI network.

### Establishment and validation of the PRG prognostic model

In order to assess the prognostic value of differentially expressed PRGs, we further conducted univariate and multivariate Cox regression analysis using the “*survival*” R package and hub PRGs were selected to construct the PRG prognostic model. The risk scores were calculated by the following formula: Risk Score = Σ_i_ X_i_×Y_i_ (X: coefficients, Y: gene expression level) and expression level was centralized and standardized. A total of 151 CLL samples were divided into high- and low-risk subgroups according to the median risk score of the PRG prognostic model and OS was compared *via* Kaplan–Meier analysis. The “*FactoMineR*” and “*factoextra*” package were used to perform PCA and “*survival*”, “*survminer*”, and “*survivalROC*” R packages were employed to perform a 3-year, 5-year, and 10-year OS ROC curve analysis. For the validation of the established model, 101 CLL samples with TTFT among 151 samples and 130 CLL patients in another cohort were employed. The expression of PRGs was also normalized and the risk scores were then calculated by the formula established by the 151-CLL-sample cohort. The efficacy of the PRG prognostic model was validated by these two cohorts according to the procedures mentioned above.

### Functional analysis of the DEGs between the low- and high-risk subgroups

A total of 151 CLL samples in GSE22762 were stratified into two subgroups according to the median risk score of the PRG prognostic model. The DEGs between the low- and high-risk subgroups were screened using the “*limma*” package. Adjusted *p*-value < 0.05 was regarded as statistically significant. DEGs were used to perform Gene Ontology (GO) analysis using Metascape (https://metascape.org/gp/index.html#/main/step1); the upregulated and downregulated DEGs were used to perform Kyoto Encyclopedia of Genes and Genomes (KEGG) analysis using the “*clusterProfiler*” package. Pathways with adjusted *p* < 0.05 were considered to be of statistical significance. Gene Set Enrichment Analysis (GSEA) between high- and low-risk subgroups based on the identified gene signature was conducted *via* the “*GSEAbase*” and “*clusterProfiler*” R package and h.all.v7.4.symbols.gmt was used as a reference gene list. Pathways with adjusted *p* < 0.05 normalized enrichment score |NES| ≥ 1 were considered significant.

### Evaluation of immune pathways and tumor immune environment status

Single-sample GSEA (ssGSEA) was conducted *via* the “*GSVA*” package, and the scores of immune cell infiltration and the activity of immune-related pathways were calculated. The reference gene list is listed in **Table S2**.

### RNA extraction and real-time PCR

Total RNA was extracted from CD19+ B cells from CLL patients by Trizol Reagent (Invitrogen, Carlsbad, CA, United States) and the extracted RNA was subsequently reverse-transcribed into cDNA using SuperScript III reverse transcriptase (Invitrogen). The cDNA was subjected to SYBR Green-based real-time PCR analysis. Gene expression changes were calculated using the comparative C_t_ method and values were normalized to β-actin expression levels. The primers used in real-time PCR assays are listed in **Table S3**.

### Statistical analysis

All data analyses were performed in the R platform (v.4.0.2, https://cran.r-project.org/). The comparison of PRG expression between CLL cells and normal B cells, hub PRG expression, scores of immune cell infiltration, activity of immune-related pathways of different risk subgroups, and hub PRG expression between IGHV mutated and unmutated CLL were performed using the Wilcoxon rank-sum test. To screen out the hub PRGs, univariate and multivariate Cox regression were conducted and the Kaplan–Meier method with a two-sided log-rank test was performed by utilizing the “*survival*” and “*survminer*” R package. Correlation coefficients were computed by Pearson’s correlation analyses. *p* < 0.05 was indicated as statistical significance.

## Results

### Identification of differentially expressed PRGs between CLL cells and normal B cells

**Figure 2:**
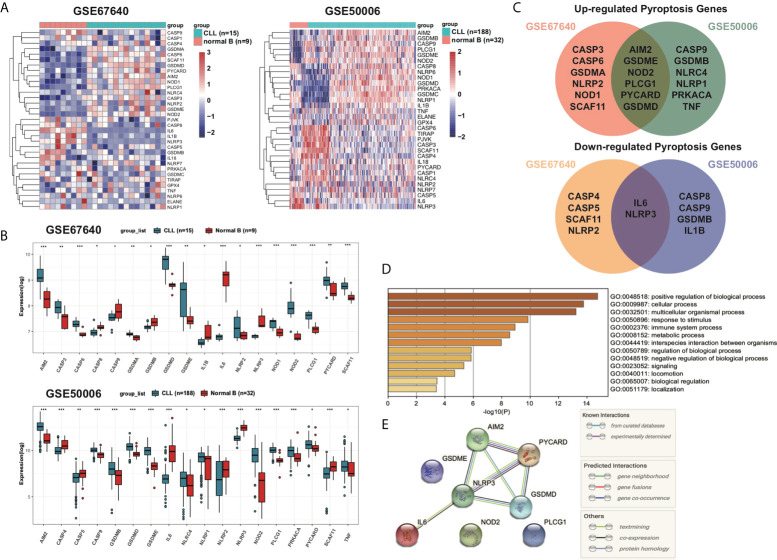
Overview of differentially expressed pyroptosis-related signatures in CLL. **(A)** Expression profiles of PRGs in CD19+ CLL and normal B cells in GEO: GSE67640 and GSE50006. Genes were clustered according to their expression. Red color represents high expression and blue color represents low expression. **(B)** Differentially expressed PRGs between CD19+ CLL and normal B cells. Adjusted *p*-values were shown as *adjusted *p* < 0.05; **adjusted *p* < 0.01; ***adjusted *p* < 0.001. **(C)** Venn of upregulated PRGs in both GSE67640 and GSE50006, and of downregulated PRGs in both GSE67640 and GSE50006. **(D)** GO analysis of differentially expressed PRGs based on the Metascape online. **(E)** PPI network showed the hub genes in the pyroptosis gene set.

The DEGs of 15 CLL samples and normal B cells from nine healthy donors were analyzed and 18 PRGs among a total of 33 PRGs were selected (adjusted *p* < 0.05). Among them, 12 genes (*AIM2, CASP3, CASP6, GSDMA, GSDMD, GSDME, NLRP2, NOD1, NOD2, PLCG1, PYCARD*, and *SCAF11*) were upregulated while six other genes (*CASP8, CASP9, GSDMB, IL-1B, IL6*, and *NLRP3*) were downregulated (**Figures 2A, B**). To validate the differentially expressed pyroptosis-related genes, another cohort including 188 CLL samples and 32 CD19+ normal B cells from healthy donors were analyzed. Six genes (*AIM2, GSDME, NOD2, PLCG1, PYCARD*, and *GSDMD*) were upregulated and two genes (*IL6* and *NLRP3*) were downregulated among CLL samples in both cohorts (**Figure 2C**). To further explore the underlying mechanisms of pyroptosis signatures in CLL, we conducted a functional analysis using Metascape. GO analysis results showed that these dysregulated PRGs were mainly enriched in response to stimulus, metabolic process, immune system process, and other biological signaling (**Figure 2D**). Therefore, these results prompt us to explore the relationship between change of TME and dysregulation of PRGs. Moreover, the protein–protein interaction (PPI) network was constructed to display the commutative relationship among dysregulated PRGs (**Figure 2E**). The minimum required interaction score for PPI analysis was set at 0.7 (high confidence), and PYCARD, AIM2, NLRP3, and GSDMD were regarded as hub genes. Among them, all the hub genes were DEGs between CLL and normal B cells.

### Development and verification of a PRG prognostic model

**Figure 3:**
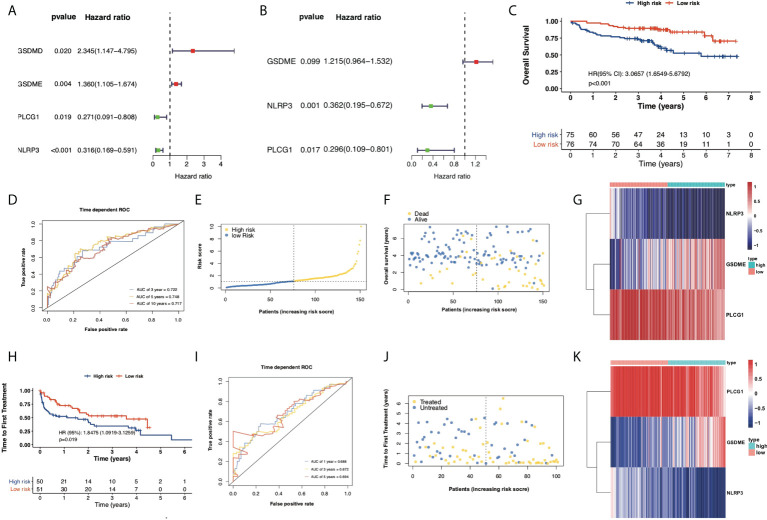
Construction of prognostic model according to multivariable Cox regression analysis. **(A)** Univariate Cox regression analysis of OS for each pyroptosis-related gene and six genes with *p* < 0.05. **(B)** Multivariate Cox regression analysis of OS for screened pyroptosis-related genes. **(C)** Kaplan–Meier plot showed OS of CLL samples in the high- and low-risk subgroups. **(D)** ROC curves showed the predictive efficiency of risk model in terms of 3-year, 5-year, and 10-year OS. **(E)** Distribution of patients based on the risk score. **(F)** Survival status of CLL samples (low risk on the left side of the dotted line and high risk on the right side of the dotted line). **(G)** Heatmap for expression of screened pyroptosis-related genes between high-risk and low-risk subgroups. **(H)** Kaplan–Meier plot showed TTFT of 101 CLL samples between high- and low-risk subgroups. **(I)** ROC curves showed the predictive efficiency of the risk model in 101 CLL samples in terms of 1-year, 3-year, and 5-year TTFT. **(J)** Treatment status of 101 CLL samples. **(K)** Heatmap for expression of screened pyroptosis-related genes between high-risk and low-risk subgroups in 101 CLL samples.

Normalized mRNA expression data with corresponding patients’ survival information were obtained from GEO. Univariate Cox regression analysis was applied to screen out the differentially expressed PRGs that were significantly associated with OS in 151 CLL patient samples. As shown in the forest plot (**Figure 3A**), four PRGs were found to be associated with prognosis. The identified prognostic PRGs (GSDMD, GSDME, NLRP3, and PLCG1) were retained for further multivariable Cox regression analysis. Among them, GSDME, NLRP3, and PLCG1 were subsequently identified as independent prognostic signatures (**Figure 3B**, **Table 2**). Therefore, a prognostic model based on the above three PRGs was established and risk scores for each patient were calculated as follows:

**Table 2:** Multivariate Cox regression analysis of signature in the 151-CLL-sample cohort.

Variable	Coef	HR (95% CI)	p-value
GSDME	0.1950	1.2153 (0.9641–1.5321)	0.0988
NLRP3	−1.0171	0.3617 (0.1946–0.6722)	0.0013
PLCG1	−1.2174	0.2960 (0.1094–0.8011)	0.0166

Coef, coefficient; HR, hazard ratio; CI, confidence interval.

Risk Score = (0.1950 × GSDME exp.) + (−1.0171 × NLRP3 exp.) + (−1.2174 × PLCG1 exp.)

Based on the median risk scores of 151 CLL samples, a high-risk subgroup (*n* = 75) and a low-risk subgroup (*n* = 76) were stratified. The median OS of the high-risk subgroup is significantly shorter than the OS of the low-risk subgroup (HR = 3.0657, *p* < 0.001, **Figure 3C**). Time-dependent ROCs were performed to evaluate the efficacy of the prognostic model, and the area under the curve (AUC) was 0.722 for 3-year survival, 0.748 for 5-year survival, and 0.717 for 10-year survival (**Figure 3D**), suggesting that the predictive model was efficient and reliable. The risk scores, survival status, and heatmap of expression profiles of these three PRGs in high- and low-risk subgroups are displayed in **Figures 3E–G**. To validate the efficacy of the established prognostic model, 101 CLL samples with TTFT among 151 CLL samples were categorized as a high-risk subgroup (*n* = 50) and a low-risk subgroup (*n* = 51) and the PRG prognostic model also showed ideal efficacy in respect of predicting TTFT (**Figures 3H–K**).

**Figure 4:**
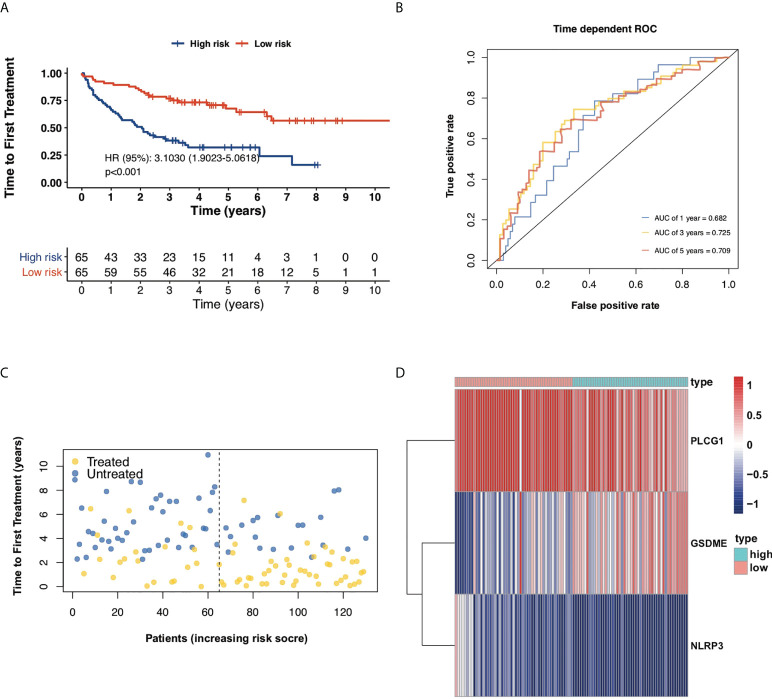
Validation of the prognostic model in respect of TTFT. **(A)** Kaplan–Meier plot showed TTFT of 130 CLL samples between high- and low-risk subgroups. **(B)** ROC curves showed the predictive efficiency of the risk model in 130 CLL samples in terms of 1-year, 3-year, and 5-year TTFT. **(C)** Treatment status of 130 CLL samples. **(D)** Heatmap for expression of screened pyroptosis-related genes between high-risk and low-risk subgroups in 130 CLL samples.

**Figure 5:**
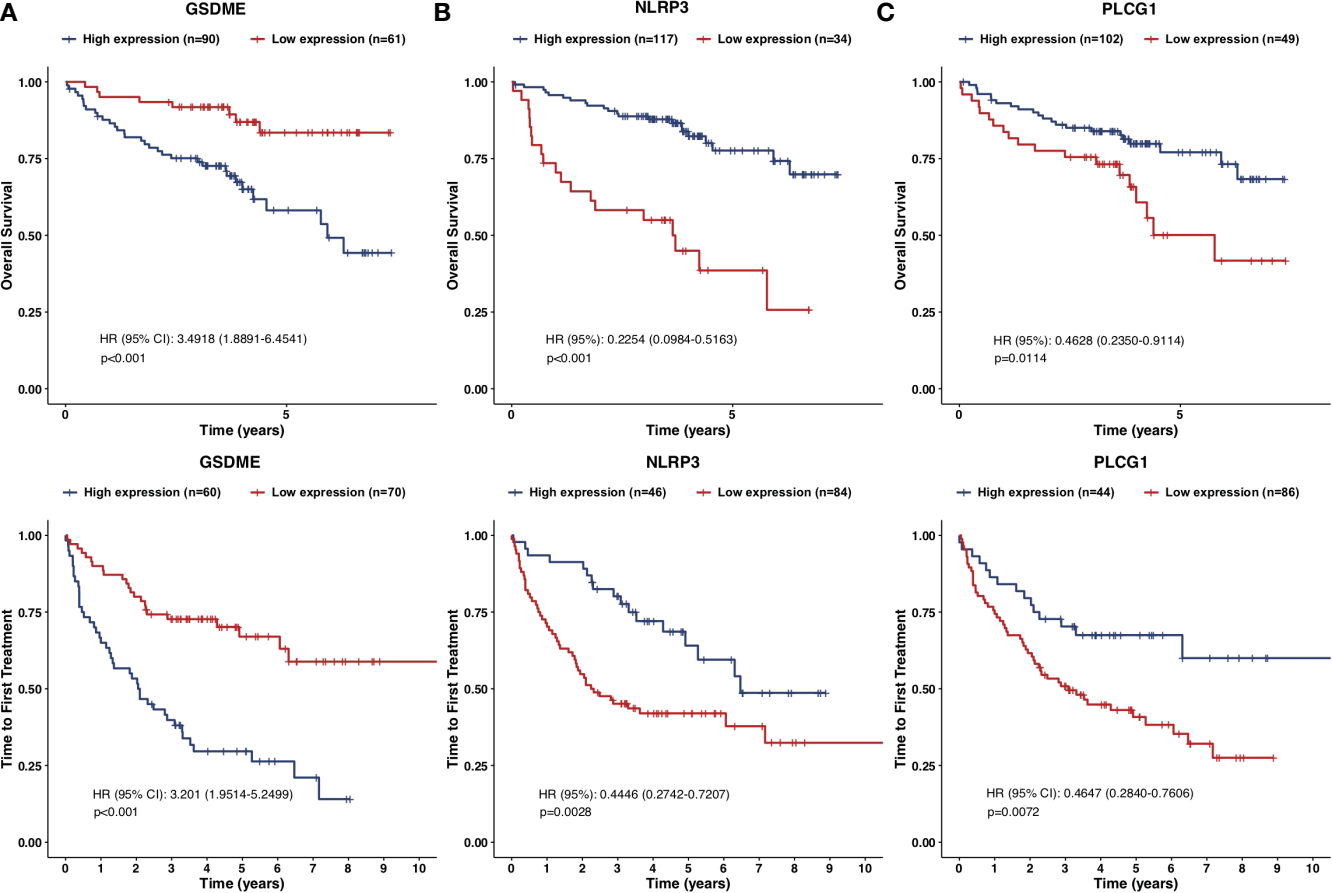
Kaplan–Meier plots of the prognostic pyroptosis-related genes. **(A–C)** Kaplan–Meier plots showed the screened pyroptosis-related genes in prognostic models with OS and TTFT: **(A)** GSDME; **(B)** NLRP3; **(C)** PLCG1.

Furthermore, another independent 130 TN CLL cohort in GES39671 was used to evaluate the reliability and accuracy of the PRG prognostic model. Kaplan–Meier plots displayed that the PRG prognostic model successfully stratified patients into low-risk (*n* = 65) and high-risk (*n* = 65) subgroups, with the median TTFT in the high-risk subgroup significantly shorter than that in the low-risk subgroup (HR = 3.1030, *p* < 0.001, **Figure 4A**). The AUCs of ROC curves were 0.682 for 1-year, 0.725 for 3-year, and 0.709 for 5-year survival (**Figure 4B**) and the PRG prognostic model also showed reliable efficacy in the validation cohort (**Figures 4C, D**). The high expression of GSDME showed poor prognosis of CLL in respect of both OS (HR = 3.4918, *p* < 0.001) and TTFT (HR = 3.201, *p* < 0.001), and the high expression of NLRP3 and PLCG1 displayed superior prognosis of CLL (**Figures 5A–C**).

### Functional analysis according to the PRG risk model

**Figure 6:**
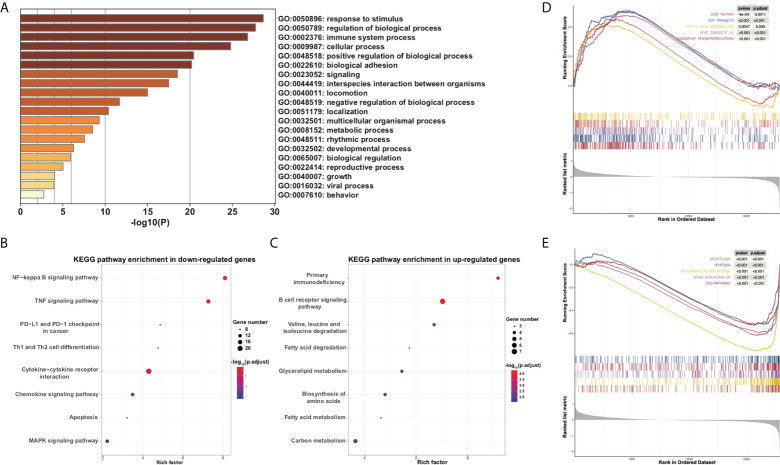
Functional analysis of DEGs between high- and low-risk subgroups. **(A)** Barplot for GO analysis of DEGs based on the Metascape online. **(B, C)** Bubbles plot for KEGG pathways of downregulated and upregulated DEGs. **(D, E)** GSEA between high- and low-risk subgroups.

To further explore the underlying functional change of PRG dysregulation, DEGs between high- and low-risk subgroups were extracted using the “*limma*” R package with the criteria adjusted *p* value < 0.05 and the absolute value of fold change (FC) ≥ 1.5. In all, 191 upregulated genes and 215 downregulated genes were identified (Table S4), and GO enrichment analysis and KEGG pathway analysis were performed. GO analysis showed that the DEGs were mainly enriched in response to stimulus, immune system process, metabolic process, rhythmic process, and other biological signaling (**Figure 6A**). KEGG analysis indicates that genes concerning NF-kappa B, apoptosis, MAPK, inflammatory pathways such as TNF, cytokine–cytokine receptor interaction, and chemokine, and immune pathways such as PD-1 and PD-L1 checkpoint, and Th1 and Th2 cell differentiation were downregulated (**Figure 6B**), while genes concerning primary immunodeficiency, B-cell receptor (BCR), and multi-substance metabolism were upregulated (**Figure 6C**). Moreover, GSEA also confirmed that metabolic pathways such as fatty acid metabolism and oxidative phosphorylation were upregulated while apoptosis and inflammatory pathways were downregulated in the high-risk subgroup of the PRG prognostic model (**Figures 6D, E**).

### Evaluation of immune status between subgroups

**Figure 7:**
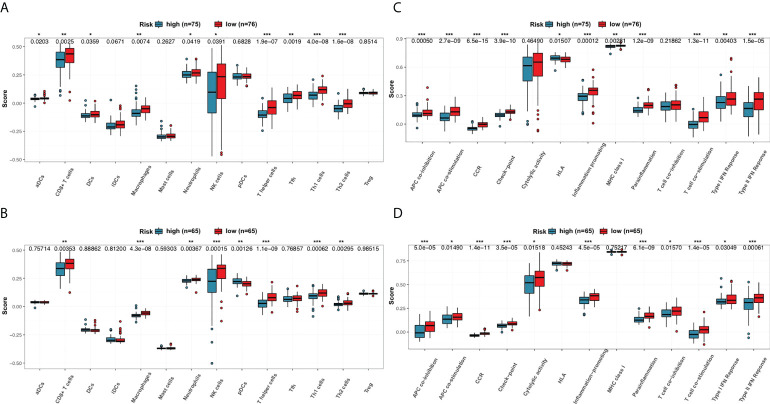
Comparison of the ssGSEA scores for immune cells and immune pathways. **(A, B)** Comparison of the enrichment scores of 14 types of immune cells between high- and low-risk subgroups in the 151-CLL-sample cohort and the 130-CLL-sample cohort. **(C, D)** Comparison of tumor immune status between high- and low-risk subgroups in the 151-CLL-sample cohort and the 130-CLL-sample cohort. *p*-values were shown as * *p* < 0.05; **p < 0.01; ***p < 0.001.

Enrichment scores of 14 types of immune cells and the activity of 13 types of immune-related pathways between high- and low-risk subgroups in both the 151-CLL-sample cohort and the 130-CLL-sample cohort were evaluated by ssGSEA. The high-risk subgroup showed generally lower levels of immune cell infiltration than in the low-risk subgroup in both cohorts, especially of CD8+ T cells, macrophages, neutrophils, natural killer (NK) cells, T helper cells, Type 1 T helper (Th1) cells, and Type 2 T helper (Th2) cells (**Figures 7A, B**). Moreover, the high-risk subgroup also displayed generally downregulated immune activity, including downregulation of antigen-presenting cell (APC) co-inhibition, APC co-stimulation, chemokine and cytokine receptor (CCR), checkpoint, inflammation promoting, parainflammation, T-cell co-stimulating, Type I interferon (IFN) response, and Type II IFN response (**Figures 7C, D**).

### Validation of the clinical prognostic value of PRG expression

**Figure 8:**
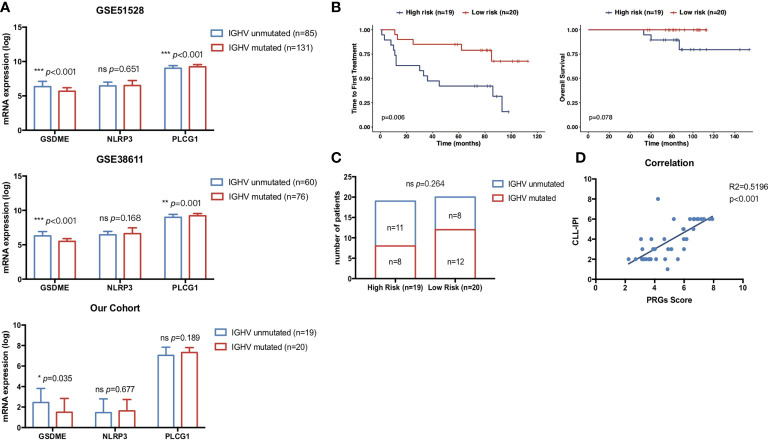
Correlation of pyroptosis-related gene expression with clinical prognostic biomarkers. **(A)** Comparison of the prognostic pyroptosis-related gene expression between 76 mutated CLL and 60 unmutated CLL samples in GSE38611. Comparison of the prognostic pyroptosis-related gene expression between 131 mutated CLL and 85 unmutated CLL samples in GSE6244. Comparison of the prognostic pyroptosis-related gene expression between 20 mutated CLL and 19 unmutated CLL samples in treatment-naïve CLL patients from our center. **(B)** Kaplan–Meier plot showed OS and TTFT of 39 treatment-naïve CLL patients in our center between high-risk (*n* = 19) and low-risk (*n* = 20) subgroups divided by PRG scores. **(C)** Distribution of IGHV status between high-risk (*n* = 19) and low-risk (*n* = 20) subgroups divided by PRG scores. **(D)** Correlation of risk scores in the pyroptosis-related gene model and CLL-IPI. *p*-values were shown as *p < 0.05; **p < 0.01; ***p < 0.001.

IGHV is one of the most well-recognized prognostic biomarkers in CLL and remained unchanged during the course of disease. Unmutated IGHV status was a key risk factor for CLL concerning both OS (34) and TTFT (35). Here, we validated the expression of our three PRGs with prognostic significance in CLL patients with unmutated IGHV and mutated IGHV status. CLL with unmutated IGHV status showed significantly higher expression of GSDME and lower expression of PLCG1 than CLL with mutated IGHV status (**Figure 8A**) in two cohorts. Furthermore, CD19+ CLL samples from 39 treatment-naïve CLL patients in our Pukou CLL center were used to validate the clinical consistency of the PRG risk model. At data cutoff on 1 March 2022, 18 of 39 patients received treatment while three patients were dead. Real-time quantitative PCR was conducted to evaluate the expression of GSDME, NLRP3, and PLCG1, and risk scores were calculated according to the formula of PRG prognostic models. GSDME showed a higher expression in IGHV unmutated CLL than in IGHV mutated CLL (*p* = 0.035), while no difference was found in NLRP3 and PLCG1 (**Figure 8A**). Risk scores according to PRG prognostic models were calculated, and our 39 patients were divided into a high-risk group (*n* = 19) and a low-risk group (*n* = 20) according to their individual PRG scores. CLL patients in the high-risk group showed significantly shorter TTFT than patients in the low-risk group (*p* = 0.006, **Figure 8B**) and showed the tendency of shorter OS than low-risk patients as well (*p* = 0.078, **Figure 8B**). The high-risk group showed more CLL patients with IGHV unmutated status than the low-risk group (**Figure 8C**). PRG risk scores also showed high consistency with CLL-IPI scores (*R* ^2^ = 0.5196, *p* < 0.001, **Figure 8D**), indicating the accordance of our established PRG risk model with well-recognized prognostic factors in clinical practice.

## Discussion

Pyroptosis, a novel form of PCD, is mediated by the gasdermin family and accompanied by inflammatory and immune responses (32). Pyroptosis was found to be closely associated with malignant tumors and might play a dual role in the pathogenesis and therapeutic mechanisms of tumors. On the one hand, the release of multiple inflammatory mediators during pyroptosis was closely associated with carcinogenesis as well as resistance to chemotherapeutic agents (32, 36). On the other hand, stimulation of pyroptosis could be a novel therapeutic target for tumor death (32). How pyroptosis functions in the development and progression of CLL remains unknown. In this study, mRNA levels of 33 currently known PRGs were compared between CLL and normal B cells, of which eight were found to be differently expressed. To further evaluate the prognostic significance of these pyroptosis-related signatures, univariate and multivariate Cox regression analysis was performed and a risk model containing three PRGs was constructed and validated. Risk scores of CLL samples were calculated according to the PRG prognostic model, and functional analysis and immune status analysis were conducted between high- and low-risk subgroups. The high-risk subgroup showed generally decreased immune cells infiltrating and a decreased level of immune-related pathways compared with the low-risk subgroup.

Our study proposed a model featuring three pyroptosis-related signatures (GSDME, NLRP3, and PLCG1) and found that they could predict OS and TTFT in CLL patients. The expression of DFNA5 (nonsyndromic hearing impairment protein 5)/GSDME was lower in most tumor cells than in normal cells due to hypermethylation (32) and DNA methylase inhibitor 5-aza-20-deoxycytosine (decitabine) could derepress GSDME *in vitro*. GSDME upregulation by decitabine may suppress tumor cell proliferation in gastric cancer, melanoma, and colorectal cancer, and inhibit the lymph node metastasis of breast cancer (37–39). Contrary to conclusions in solid tumor, the expression of GSDME was significantly upregulated in CLL and the high expression of GSDME might indicate poor prognosis in terms of OS and TTFT. This might partly be explained by the caspase-3/GSDME signal pathway, which could shift the balance between apoptosis and pyroptosis in cancer (13). Activated caspase-3 cleaves GSDME instead of PARP when GSDME is highly expressed, triggering pyroptosis rather than apoptosis. CLL showed great sensitivity and a favorable response to apoptotic pathways activation. GSDME upregulation might shift CLL programmed death from apoptosis to pyroptosis, causing drug resistance to CLL pro-apoptotic agents. Moreover, a recent study showed that during Chimeric antigen receptor (CAR) T-cell attack, cytokine-release syndrome (CRS) was a consequence of extensive pyroptosis caused by activation of both GSDME and caspase-3 and CRS was blocked by knocking out GSDME in mice (40). NLRP3 (NOD-, LRR-, and pyrin domain-containing protein 3) is a key modulator of the formation and activation of NLRP3 inflammasome. NLRP3 inflammasome activation results in the caspase 1-dependent release of pro-inflammatory cytokines IL-1β, IL-18, and gasdermin D-mediated pyroptosis (41). Studies showed that the expression of NLRP3 in hepatocellular carcinoma was significantly downregulated and NLRP3 deficiency was associated with advanced clinical stage and poor pathological differentiation (42). When it comes to CLL, consistent with what we found, downregulation of NLRP3 was shown in CLL and the low expression of NLRP3 indicated poor prognosis. Phospholipase Cgamma 1 (PLCG1), in cooperation with PLCG2, was implicated as a critical mediator of B-cell receptor pathway activation and CLL pathogenesis (43). PLCG1 downregulation or pharmacological inhibition of PLCG1 phosphorylation was reported to hinder CD47-mediated killing of CLL (44). Other than CLL, reduced PLCG1 expression was also found to be associated with inferior survival for myelodysplastic syndromes (MDS). Through the regulation of the JAK2-STAT5 pathway, PLCG1 is involved in cell survival, cell proliferation, and cell cycle progression. PLCG1 also mediates GSDMD activity and enables caspase-independent pyroptosis (45). In summary, GSDME in the prognostic model was proven to be a pyroptosis promoter while NLRP3 and PLCG1 were identified as pyroptosis executors. However, how these genes function during pyroptosis in CLL remains to be further explored.

The role of pyroptosis in cancer has not been fully understood, let alone CLL. Certain similarities and crossovers in mechanisms between pyroptosis and apoptosis have been found, but how multiple modes of cell death coexist and interact with each other remains largely unknown. Herein, GSDME and PLCG1 in our PRG prognostic model are also key regulators in apoptotic pathways (13, 44). To understand the underlying mechanism of pyroptosis in CLL, DEGs between high- and low-risk groups were analyzed and functional analysis was conducted. Based on the results of GO, KEGG, and GSEA, pathways concerning immune and inflammatory response were significantly downregulated while pathways concerning metabolism and BCR signaling were significantly upregulated, suggesting that dysregulation of pyroptosis in the high-risk group might hinder inflammatory response. Analysis of immune cell infiltration and activity of immune-related pathways also validate the conclusion that the poor outcome of high-risk CLL patients might be partly attributed to decreased antitumor immunity.

Due to the small number of current studies exploring the role of pyroptosis in CLL, our study systematically screened the prognostic PRGs and identified three genes that are associated with OS and TTFT in CLL patients. However, the limitations of our study should be pointed out. First of all, how these prognostic PRGs interact with each other remains to be further investigated and is not clarified in our risk model. Second, the underlying mechanism of these PRGs in the process of CLL occurrence and progression needs to be explored *via* molecular experiments. Last but not least, the depiction of the CLL immune microenvironment needs to be validated. Advanced technologies like single-cell RNA sequencing should be applied to mine pyroptosis-related immune environment features.

In summary, our study screened out PRGs differentially expressing between CLL and normal B cells. The risk score generated from the established prognostic model based on three PRGs was of high efficacy for predicting OS and TTFT in two independent CLL cohorts. Functional and immune status analysis between high- and low-risk CLL concluded that dysregulation of pyroptosis in high-risk CLL patients might lead to decreased antitumor immunity. Our study explored the prognostic value of PRGs in predicting OS and TTFT in CLL patients and shed light on further studies on the underlying mechanism including the CLL immune microenvironment.

## Data availability statement

The original contributions presented in the study are included in the article/**Supplementary Material**. Further inquiries can be directed to the corresponding authors.

## Author contributions

HZ, JL, and WX conceived and designed the experiments. YS and RJ performed the experiments and conducted data analysis. YS and HZ wrote the manuscript. YM, SQ, WW, YX, LW, LF, and HJ supervised the research. All authors contributed to the article and approved the submitted version.

## Funding

This study was supported by the National Natural Science Foundation of China (Grant No. 82170166, 82100207, 81970146, and 81900167), the National Science Foundation of China International Cooperation and Exchange Program (Grant No. 81720108002), Translational Research Grant of NCRCH (2020ZKZB01), and the Six Talent Peaks Project in Jiangsu Province 2019 (Grant No. WSN-001).

## Conflict of interest

The authors declare that the research was conducted in the absence of any commercial or financial relationships that could be construed as a potential conflict of interest.

## Publisher’s note

All claims expressed in this article are solely those of the authors and do not necessarily represent those of their affiliated organizations, or those of the publisher, the editors and the reviewers. Any product that may be evaluated in this article, or claim that may be made by its manufacturer, is not guaranteed or endorsed by the publisher.

## References

[1] Hallek M . Chronic lymphocytic leukemia: 2017 update on diagnosis, risk stratification, and treatment. Am J Hematol (2017) 92(9):946–65. doi: 10.1002/ajh.24826 28782884

[2] International CLL-IPI working group . An international prognostic index for patients with chronic lymphocytic leukaemia (Cll-ipi): A meta-analysis of individual patient data. Lancet Oncol (2016) 17(6):779–90. doi: 10.1016/s1470-2045(16)30029-8 27185642

[3] Sha YQ Shen H Wu W Xia Y Miao Y Cao L . [Comparison of four prognostic scoring system in patients with early asymptomatic chronic lymphocytic leukemia patients]. Zhonghua xue ye xue za zhi = Zhonghua xueyexue zazhi (2021) 42(10):834–9. doi: 10.3760/cma.j.issn.0253-2727.2021.10.007 PMC8607014 34788923

[4] Zhu HY Wang L Qiao J Zou YX Xia Y Wu W . [Prognostic significance of cll-ipi for Chinese patients with chronic lymphocytic leukemia]. Zhonghua xue ye xue za zhi = Zhonghua xueyexue zazhi (2018) 39(5):392–7. doi: 10.3760/cma.j.issn.0253-2727.2018.05.009 PMC7342903 29779348

[5] Patel K Pagel JM . Current and future treatment strategies in chronic lymphocytic leukemia. J Hematol Oncol (2021) 14(1):69. doi: 10.1186/s13045-021-01054-w 33902665 PMC8074228

[6] Eichhorst B Hallek M . Prognostication of chronic lymphocytic leukemia in the era of new agents. Hematol Am Soc Hematol Educ Program (2016) 2016(1):149–55. doi: 10.1182/asheducation-2016.1.149 PMC6142472 27913474

[7] Xu W Yang S Zhou K Pan L Li Z Zhou J . Treatment of Relapsed/Refractory chronic lymphocytic Leukemia/Small lymphocytic lymphoma with the btk inhibitor zanubrutinib: Phase 2, single-arm, multicenter study. J Hematol Oncol (2020) 13(1):48. doi: 10.1186/s13045-020-00884-4 32393328 PMC7216400

[8] Stilgenbauer S Eichhorst B Schetelig J Coutre S Seymour JF Munir T . Venetoclax in relapsed or refractory chronic lymphocytic leukaemia with 17p deletion: A multicentre, open-label, phase 2 study. Lancet Oncol (2016) 17(6):768–78. doi: 10.1016/s1470-2045(16)30019-5 27178240

[9] Stilgenbauer S Eichhorst B Schetelig J Hillmen P Seymour JF Coutre S . Venetoclax for patients with chronic lymphocytic leukemia with 17p deletion: Results from the full population of a phase ii pivotal trial. J Clin Oncol Off J Am Soc Clin Oncol (2018) 36(19):1973–80. doi: 10.1200/jco.2017.76.6840 29715056

[10] Fischer K Al-Sawaf O Bahlo J Fink AM Tandon M Dixon M . Venetoclax and obinutuzumab in patients with cll and coexisting conditions. New Engl J Med (2019) 380(23):2225–36. doi: 10.1056/NEJMoa1815281 31166681

[11] Roberts AW Wei AH Huang DCS . Bcl2 and Mcl1 inhibitors for hematologic malignancies. Blood (2021) 138(13):1120–36. doi: 10.1182/blood.2020006785 34320168

[12] Chipuk JE Moldoveanu T Llambi F Parsons MJ Green DR . The bcl-2 family reunion. Mol Cell (2010) 37(3):299–310. doi: 10.1016/j.molcel.2010.01.025 20159550 PMC3222298

[13] Jiang M Qi L Li L Li Y . The caspase-3/Gsdme signal pathway as a switch between apoptosis and pyroptosis in cancer. Cell Death Discovery (2020) 6:112. doi: 10.1038/s41420-020-00349-0 33133646 PMC7595122

[14] Wang Y Gao W Shi X Ding J Liu W He H . Chemotherapy drugs induce pyroptosis through caspase-3 cleavage of a gasdermin. Nature (2017) 547(7661):99–103. doi: 10.1038/nature22393 28459430

[15] Tang R Xu J Zhang B Liu J Liang C Hua J . Ferroptosis, necroptosis, and pyroptosis in anticancer immunity. J Hematol Oncol (2020) 13(1):110. doi: 10.1186/s13045-020-00946-7 32778143 PMC7418434

[16] Shi J Zhao Y Wang K Shi X Wang Y Huang H . Cleavage of gsdmd by inflammatory caspases determines pyroptotic cell death. Nature (2015) 526(7575):660–5. doi: 10.1038/nature15514 26375003

[17] Kayagaki N Stowe IB Lee BL O'Rourke K Anderson K Warming S . Caspase-11 cleaves gasdermin d for non-canonical inflammasome signalling. Nature (2015) 526(7575):666–71. doi: 10.1038/nature15541 26375259

[18] Julien O Wells JA . Caspases and their substrates. Cell Death differentiation (2017) 24(8):1380–9. doi: 10.1038/cdd.2017.44 PMC5520456 28498362

[19] Crawford ED Wells JA . Caspase substrates and cellular remodeling. Annu Rev Biochem (2011) 80:1055–87. doi: 10.1146/annurev-biochem-061809-121639 21456965

[20] Guo J Xu B Han Q Zhou H Xia Y Gong C . Ferroptosis: A novel anti-tumor action for cisplatin. Cancer Res Treat (2018) 50(2):445–60. doi: 10.4143/crt.2016.572 PMC5912137 28494534

[21] Ursic K Kos S Kamensek U Cemazar M Scancar J Bucek S . Comparable effectiveness and immunomodulatory actions of oxaliplatin and cisplatin in electrochemotherapy of murine melanoma. Bioelectrochemistry (Amsterdam Netherlands) (2018) 119:161–71. doi: 10.1016/j.bioelechem.2017.09.009 29024870

[22] Zhang CC Li CG Wang YF Xu LH He XH Zeng QZ . Chemotherapeutic paclitaxel and cisplatin differentially induce pyroptosis in A549 lung cancer cells. Via Caspase-3/Gsdme Activation. Apoptosis an Int J programmed Cell Death (2019) 24(3-4):312–25. doi: 10.1007/s10495-019-01515-1 30710195

[23] Yu P Wang HY Tian M Li AX Chen XS Wang XL . Eukaryotic elongation factor-2 kinase regulates the cross-talk between autophagy and pyroptosis in doxorubicin-treated human melanoma cells *in vitro* . Acta pharmacologica Sin (2019) 40(9):1237–44. doi: 10.1038/s41401-019-0222-z PMC6786479 30914761

[24] Zhang Z Zhang Y Xia S Kong Q Li S Liu X . Gasdermin e suppresses tumour growth by activating anti-tumour immunity. Nature (2020) 579(7799):415–20. doi: 10.1038/s41586-020-2071-9 PMC7123794 32188940

[25] Zhou Z He H Wang K Shi X Wang Y Su Y . Granzyme a from cytotoxic lymphocytes cleaves gsdmb to trigger pyroptosis in target cells. Sci (New York NY) (2020) 368(6494):eaaz7548. doi: 10.1126/science.aaz7548 32299851

[26] Filarsky K Garding A Becker N Wolf C Zucknick M Claus R . Krüppel-like factor 4 (Klf4) inactivation in chronic lymphocytic leukemia correlates with promoter DNA-methylation and can be reversed by inhibition of notch signaling. Haematologica (2016) 101(6):e249–53. doi: 10.3324/haematol.2015.138172 PMC5013946 27081174

[27] Herold T Jurinovic V Metzeler KH Boulesteix AL Bergmann M Seiler T . An eight-gene expression signature for the prediction of survival and time to treatment in chronic lymphocytic leukemia. Leukemia (2011) 25(10):1639–45. doi: 10.1038/leu.2011.125 21625232

[28] Chuang HY Rassenti L Salcedo M Licon K Kohlmann A Haferlach T . Subnetwork-based analysis of chronic lymphocytic leukemia identifies pathways that associate with disease progression. Blood (2012) 120(13):2639–49. doi: 10.1182/blood-2012-03-416461 PMC3460686 22837534

[29] Maura F Cutrona G Mosca L Matis S Lionetti M Fabris S . Association between gene and mirna expression profiles and stereotyped subset 4 b-cell receptor in chronic lymphocytic leukemia. Leukemia lymphoma (2015) 56(11):3150–8. doi: 10.3109/10428194.2015.1028051 25860243

[30] Fabris S Mosca L Cutrona G Lionetti M Agnelli L Ciceri G . Chromosome 2p gain in monoclonal b-cell lymphocytosis and in early stage chronic lymphocytic leukemia. Am J Hematol (2013) 88(1):24–31. doi: 10.1002/ajh.23340 23044996

[31] Leek JT Johnson WE Parker HS Jaffe AE Storey JD . The sva package for removing batch effects and other unwanted variation in high-throughput experiments. Bioinf (Oxford England) (2012) 28(6):882–3. doi: 10.1093/bioinformatics/bts034 PMC3307112 22257669

[32] Xia X Wang X Cheng Z Qin W Lei L Jiang J . The role of pyroptosis in cancer: Pro-cancer or pro-"Host"? Cell Death Dis (2019) 10(9):650. doi: 10.1038/s41419-019-1883-8 31501419 PMC6733901

[33] Wang YY Liu XL Zhao R . Induction of pyroptosis and its implications in cancer management. Front Oncol (2019) 9:971. doi: 10.3389/fonc.2019.00971 31616642 PMC6775187

[34] Parikh SA Strati P Tsang M West CP Shanafelt TD . Should ighv status and fish testing be performed in all cll patients at diagnosis? A systematic review and meta-analysis. Blood (2016) 127(14):1752–60. doi: 10.1182/blood-2015-10-620864 26841802

[35] Hu B Patel KP Chen HC Wang X Luthra R Routbort MJ . Association of gene mutations with time-to-First treatment in 384 treatment-naive chronic lymphocytic leukaemia patients. Br J haematology (2019) 187(3):307–18. doi: 10.1111/bjh.16042 31243771

[36] Thi HTH Hong S . Inflammasome as a therapeutic target for cancer prevention and treatment. J Cancer Prev (2017) 22(2):62–73. doi: 10.15430/jcp.2017.22.2.62 28698859 PMC5503217

[37] Rogers C Erkes DA Nardone A Aplin AE Fernandes-Alnemri T Alnemri ES . Gasdermin pores permeabilize mitochondria to augment caspase-3 activation during apoptosis and inflammasome activation. Nat Commun (2019) 10(1):1689. doi: 10.1038/s41467-019-09397-2 30976076 PMC6459836

[38] Croes L de Beeck KO Pauwels P Vanden Berghe W Peeters M Fransen E . Dfna5 promoter methylation a marker for breast tumorigenesis. Oncotarget (2017) 8(19):31948–58. doi: 10.18632/oncotarget.16654 PMC5458261 28404884

[39] Zhang Z Zhang Y Lieberman J . Lighting a fire: Can we harness pyroptosis to ignite antitumor immunity? Cancer Immunol Res (2021) 9(1):2–7. doi: 10.1158/2326-6066.Cir-20-0525 33397791 PMC7789047

[40] Liu Y Fang Y Chen X Wang Z Liang X Zhang T . Gasdermin e-mediated target cell pyroptosis by car T cells triggers cytokine release syndrome. Sci Immunol (2020) 5(43):eaax7969. doi: 10.1126/sciimmunol.aax7969 31953257

[41] Swanson KV Deng M Ting JP . The Nlrp3 inflammasome: Molecular activation and regulation to therapeutics. Nat Rev Immunol (2019) 19(8):477–89. doi: 10.1038/s41577-019-0165-0 PMC7807242 31036962

[42] Wei Q Mu K Li T Zhang Y Yang Z Jia X . Deregulation of the Nlrp3 inflammasome in hepatic parenchymal cells during liver cancer progression. Lab investigation; J Tech Methods Pathol (2014) 94(1):52–62. doi: 10.1038/labinvest.2013.126 24166187

[43] Marshall AJ Niiro H Yun TJ Clark EA . Regulation of b-cell activation and differentiation by the phosphatidylinositol 3-kinase and phospholipase cgamma pathway. Immunol Rev (2000) 176:30–46. doi: 10.1034/j.1600-065x.2000.00611.x 11043766

[44] Martinez-Torres AC Quiney C Attout T Boullet H Herbi L Vela L . Cd47 agonist peptides induce programmed cell death in refractory chronic lymphocytic leukemia b cells *Via* Plcγ1 activation: Evidence from mice and humans. PloS Med (2015) 12(3):e1001796. doi: 10.1371/journal.pmed.1001796 25734483 PMC4348493

[45] Kang R Zeng L Zhu S Xie Y Liu J Wen Q . Lipid peroxidation drives gasdermin d-mediated pyroptosis in lethal polymicrobial sepsis. Cell Host Microbe (2018) 24(1):97–108.e4. doi: 10.1016/j.chom.2018.05.009 29937272 PMC6043361

